# Comparing incomparables: commentary on “measuring the frequency of inner-experience characteristics”

**DOI:** 10.3389/fpsyg.2024.1361110

**Published:** 2024-02-19

**Authors:** Thomas M. Brinthaupt, Alain Morin, Bob Uttl

**Affiliations:** ^1^Department of Psychology, Middle Tennessee State University, Murfreesboro, TN, United States; ^2^Department of Psychology, Mount Royal University, Calgary, AB, Canada

**Keywords:** inner speech, self-talk, individual differences, self-report measures, frequency, Descriptive Experience Sampling, inner-experiences, sample size

## Introduction

Hurlburt et al. (2022) compared self-talk frequency using three methods: (a) a questionnaire asking participants to rate how often they talk to themselves in specific situations (Self-Talk Scale, STS; Brinthaupt et al., 2009), (b) a questionnaire (The Nevada Inner Experience Questionnaire, NIEQ; Heavey et al., 2019) asking participants to rate how often they engage in inner speech and (c) the Descriptive Experience Sampling technique (DES; see Heavey and Hurlburt, 2008)—an interview-based technique probing inner experiences occurring in participants' natural environment. Hurlburt et al. (2022) concluded that “… estimates of inner-experience frequency produced by questionnaires [STS and NIEQ] and DES are irreconcilably discrepant…” (p. 559) and that questionnaires inflate the frequencies of self-reported inner experiences and self-talk. In what follows, we demonstrate that Hurlburt et al.'s conclusions are unwarranted, principally because the authors compared incomparables, apples to oranges.

## Brief overview of the DES, STS, and NIEQ

DES consists of sampling participants' inner experiences by randomly beeping them during specific time windows each day for several days in their natural environment and asking them to note what they were experiencing right before the beep. Participants are later queried about their self-reports, typically within 24 h, by a set of interviewers. One of the interviewers writes down what the participant told them and then circulates this description with others to comment on and to correct, again, typically within 24 h. After 4 days of sampling is completed, all interviewers met to review and refine the previous interview descriptions. Thus, what is coded is not directly the participants' experience but the participants' recollection, then the interviewers' interpretation, and re-interpretation of participants' recollection guided by the interviewers themselves. DES investigators continuously discuss their interpretations with the participant, and the final coding into “experience types” is done independently and validated. Notably, DES does not systematically ask nor explain to participants what various inner experiences are.

In contrast, the STS (Brinthaupt et al., 2009) includes 16 statements which respondents use to rate how often they talk to themselves in specific situations using a 5-point scale ranging from 1 = never to 5 = very often. STS scores are respondents' subjective ratings of how often they talk to themselves when those situations occur. STS scores do not give any indication as to how many times per hour or what proportion of time participants talk to themselves in general. The calculation and analysis of STS subscale scores provide measures of how often respondents use self-talk for the four different functions. Researchers typically examine those four subscales (functions) rather than estimating how often people talk to themselves in general. In other words, the STS measures the frequency of use of specific functions or reasons for talking to oneself and is not an estimate of how often people talk to themselves in their everyday experiences. To our knowledge, no one (other than Hurlburt et al.) has tried to interpret total or subscale STS scores as measures of absolute or relative self-talk frequency.

Heavey et al. (2019) created a brief measure of inner experiences designed to assess subjective frequency of inner speech, inner seeing, unsymbolized thinking, sensory awareness, and feelings. The NIEQ consists of 10 items, with two items referring to each characteristic. The inner speech items are “How frequently do you talk to yourself in your inner voice?” (rated using a 0–100 visual analog scale ranging from Never to Always—a subjective frequency scale) and “Generally speaking, what portion of your inner experience is in inner speech (thinking in words)?” (rated on a None to All 0–100 visual analog scale). An average of the two items' ratings provides the inner speech subscale score. This score therefore represents a combination of respondents' assessment of their typical subjective inner speech frequency and the proportion of their inner experience that takes the form of inner speech. Again, NIEQ average scores do not give any indication as to how many times per hour or what proportion of time participants talk to themselves in general.

## Apples to oranges comparison problem

Hurlburt et al. (2022) use STS scores to assess participants' typical inner speech and self-talk and compare those scores to the DES results. However, the two methods aim to gather incomparable data. The STS measures self-talk *when specific situations occur* using a subjective frequency scale and, as the result, the STS scores do not say anything at all about how often people talk to themselves in general or what proportion of their daily experience involves self-talk.

In contrast, the authors' DES data show whether participants are talking to themselves at or around the specific moment that they were beeped, not in response to a specific situation. As the result, DES data, unencumbered by various problems with DES technique, would yield absolute frequencies of inner speech and self-talk, that is, how many times per hour or what proportion of time participants engaged in inner speech and/or self-talk. These absolute frequencies cannot be compared to the STS data. Hurlburt et al. make no argument and provide no evidence that talking to oneself is a “random” activity or inner experience. We argue that self-talk is almost always produced in response to or in reaction to a specific event or environmental stimulus. As such, the DES approach will inevitably underestimate people's frequency of self-talk because it does not directly examine situations that would cause or encourage a person to engage in inner speech. It appears that the only instances detected by the DES approach are those that correspond to situations occurring randomly that induce inner speech.

Similarly, the NIEQ does not measure how often people talk to themselves or what proportion of time people talk to themselves. Neither question about inner speech on the NIEQ can provide any insight into absolute frequency of inner speech engaged in by respondents. The first question only asks about how often participants talk to themselves in their “inner voice.” The second question only asks about the proportion of participants' inner experiences that were inner speech. In their NIEQ paper (Heavey et al., 2019, Table 2), the “portion” means reported for the five kinds of inner experience range from 35–69% for each. In other words, they do not total 100% as we would expect if respondents were making a proportionate judgment (i.e., inner speech % compared to the other four kinds of inner experience). Thus, it is unclear what to make of data based on this second question.

## Methodological and statistical problems

The DES suffers from several major methodological problems rendering its scores largely uninterpretable. First, the DES does not systematically explain to participants what various inner experiences are. This avoidance of “closed-beginning” (content-specifying) questions such as, “Were you innerly arguing at the moment of the beep?” (Hurlburt et al., 2022, Supplementary material, p. 5) is considered a feature of the method. However, people tend to report only observations that they see as relevant to the circumstances. If people see something as irrelevant, they rarely report it. Uttl and Kisinger (2010) demonstrated this in a simple experiment. Participants in their study watched car accident videos and minutes later were asked to recall everything they could remember about each accident. Although participants rarely reported observations irrelevant to why the accidents happened (e.g., no rain/dry road), they were almost 100% accurate when they were directly asked whether an event (e.g., rain/wet road) was present or absent during the accident. Accordingly, it is better to ask whether people talk to themselves in response to specific situations than to see if inner speech occurred during some random time.

Second, DES data are dependent upon participants' memories as well as the interviewers' guidance of that recollection. This means that whether participants mention that they talked to themselves at the time of the beep depends on (a) whether they actually did talk to themselves, (b) whether they had sufficiently detailed memory of their inner experiences up to 24 h earlier, (c) whether they realized and understood at any point that their self-talk was of interest to the interviewers, and (d) whether the interviewers focused on participants' retrospective accounts of specific instances of self-talk. DES data further depend on the interviewers' interpretation of the participants' recollections.

Close examination of Hurlburt et al. (2022) studies also shows that their samples are extraordinarily small to allow any valid conclusions, even if the DES, NIEQ, and STS data measured proportions of the time people have various inner experiences (which they do not). In addition, their statistical analyses are problematic. For example, the distribution of DES vs. STS percentages are not normal, with both showing excessive skewness—DES data suffering from a floor effect and STS data suffering from a ceiling effect (see Supplementary Figure S2, p. 60)—and as the result, parametric statistics such as *t*-tests used are not appropriate.

Results obtained on such small samples lack precision, have low statistical power, result in inflated discovery rate as well as low generalizability, and are, in general, uninterpretable (Ioannidis, 2005). Hurlburt et al. (2022) themselves dismissed their small samples results as lacking precision and being invalid; when interpreting the correlations between NIEQ and DES, Hurlburt et al. wrote (in Supplementary Box S18, p. 55) verbatim: “... These are across-participant correlations, and therefore the degrees of freedom are small with resulting large standard errors [poor precision]. As a result, we did not present these results in the main paper, leaving it to the reader of the Supplemental material to decide how seriously to take them.... All these correlations were very close to zero; the largest correlation (for inner seeing, *r* = 0.29, *p* = 0.276) was not significant even without adjusting for multiple correlations.”

Most critically, Hurlburt et al. (2022) state: “It can be seen that the correlation between NIEQ and DES measures of inner-speaking are close to zero and similar across the three studies... Thus, it is not merely the case that NIEQ questionnaire scores routinely overestimate the absolute magnitude of DES sampling frequencies...; NIEQ questionnaire scores also have little or no relationship to the relative magnitude of DES sampling frequencies of the 5FP [5 Frequent Phenomena]” (Supplemental material, p. 58).

Thus, if correlations between NIEQ and DES scores are truly zero (rather than artifacts of small samples), the NIEQ and DES—according to Hurlburt et al.'s own data – do NOT measure the same nor similar constructs. In turn, the comparison of NIEQ and DES mean scores amounts to comparison of apples and oranges exactly as we would expect from the analysis of what each of the three measures aims to measure. It is simply unreasonable to make strong claims based on STS, NIEQ, and DES mean scores and to dismissing zero correlations among these measures indicating that these measures do not measure the same constructs because of small samples used to produce these data.

However, with these small sample sizes, there is no point to even calculate correlations as confidence intervals on any such correlations are so wide as to make their estimates useless. If the correlation between the DES and NIEQ was found to be 0, with a sample size of 12, we can be 95% confident that the population correlation is between −0.57 to +0.57—not very useful given that zero correlations indicate no relationship between the two sets of scores whereas 0.57 indicates moderately strong relationship between the two sets of scores. It is worth noting that their own DES results (Heavey and Hurlburt, 2008) are different from the ones reported in Hurlburt et al. (2022). This supports our concerns about sample size influences and lack of power (see also Supplementary Box S17).

## Conclusions

The three methods used by Hurlburt et al. (2022) measure different things and their scores cannot be compared. The STS asks about self-talk in specific situations. The NIEQ asks about inner speech in general and what proportions of inner experiences are inner speech. DES aims to discover absolute frequency of inner speech and self-talk, that is, proportion of times participants engage in inner speech or self-talk at random times during the day. The STS and NIEQ use relative subjective frequency scales whereas the DES is using counts per specific time period. Finally, the STS subjective frequency scale end points (“Very Often”) are not at all equivalent to the NIEQ subjective frequency scale endpoints (“Always”). Simply put: (a) “very often” is not equal to “always,” (b) “always” is not necessarily equal to 100% of the time, and (c) saying I “always” talk to myself is obviously different from saying whenever I accidentally hit my finger with my hammer, I “always” talk to myself.

Hurlburt et al. offer four possible explanations for the lack of correspondence between the measures they used. One explanation is that the differences they found are “merely a difference in the point of view between questionnaires and DES” (p. 566). We argue, for several reasons we have noted, that this is the most likely explanation for their results as opposed to their favored interpretation of “an overestimation of the frequency of actual phenomena by questionnaires” (p. 566). In addition, because no one to our knowledge has tried predicting behavior from DES, the evidence is lacking that DES provides a better measure than a questionnaire when explaining externally observable behavior. Finally, the questionnaire data do not provide insight into why DES and other kinds of experience sampling would be so different.

The question of how often people talk to themselves during the flow of their natural environment is, for sure, an interesting and important research topic. However, a measure that does not assess this phenomenon (such as the STS) cannot be used to measure it; it is invalid for that purpose. One can hardly expect the percentage of time people carry umbrellas on any given day to be the same as the percentage of time people carry umbrellas on rainy days. Whereas there are good reasons to be concerned about the validity of self-report measures of inner speech (e.g., Uttl et al., 2011; Brinthaupt and Morin, 2020), the failure of two of those measures to correspond to the kind of DES data reported in the Hurlburt et al. (2022) is not one of them. The authors are comparing incomparables.

## Author contributions

TB: Conceptualization, Supervision, Writing—original draft, Writing—review & editing. AM: Conceptualization, Writing—original draft, Writing—review & editing. BU: Conceptualization, Writing—original draft, Writing—review & editing.
